# Sperm Dysfunction in the Testes and Epididymides due to Overweight and Obesity Is Not Caused by Oxidative Stress

**DOI:** 10.1155/2022/3734572

**Published:** 2022-10-10

**Authors:** Lorena Ruiz-Valderrama, Jaqueline Posadas-Rodríguez, Herlinda Bonilla-Jaime, Maria del Rosario Tarragó-Castellanos, Humberto González-Márquez, Isabel Arrieta-Cruz, Leticia González-Núñez, Arturo Salame-Méndez, Ahiezer Rodríguez-Tobón, José Guadalupe Morales-Méndez, Edith Arenas-Ríos

**Affiliations:** ^1^Doctorado en Ciencias Biológicas y de la Salud, Universidad Autónoma Metropolitana, Ciudad y Estado de México, Mexico; ^2^Maestría en Biología de la Reproducción Animal, Universidad Autónoma Metropolitana, Iztapalapa, Ciudad de México 09340, Mexico; ^3^Departamento de Biología de La Reproducción, Universidad Autónoma Metropolitana, Iztapalapa, Ciudad de México 09340, Mexico; ^4^Departamento de Ciencias de La Salud, Universidad Autónoma Metropolitana, Iztapalapa, Ciudad de México 09340, Mexico; ^5^Departamento de Investigación Básica, Instituto Nacional de Geriatría, Ciudad de México 10200, Mexico; ^6^Departamento de Biología, Universidad Autónoma Metropolitana, Iztapalapa, Ciudad de México 09340, Mexico; ^7^Ciencias Físicas, Universidad Autónoma Metropolitana, Iztapalapa, Ciudad de México 09340, Mexico

## Abstract

Obesity is a condition that has been linked to male infertility. The current hypothesis regarding the cause of infertility is that sperm are highly sensitive to reactive oxygen species (ROS) during spermatogenesis in the testes and transit through the epididymides, so the increase in ROS brought on by obesity could cause oxidative stress. The aim of this study was to evaluate whether the activity of the enzymes catalase (CAT), superoxide dismutase (SOD), and glutathione peroxidase (GPX) is capable of counteracting oxidative stress in sperm. The male Wistar rat was used as an overweight and obesity model, and analysis of fertility in these groups was carried out including the control group. Serum testosterone levels were determined, and the scrotal fat, testes, and epididymides were extracted. The epididymides were separated ini0 3 principal parts (caput, corpus, and cauda) before evaluating sperm viability, sperm morphology, damage to desoxyribonucleic acid of the sperm, and ROS production. The protein content and specific activity of the three enzymes mentioned above were evaluated. Results showed a gain in body weight and scrotal fat in the overweight and obese groups with decreased parameters for serum testosterone levels and sperm viability and morphology. Fertility was not greatly affected and no DNA integrity damage was found, although ROS in the epididymal sperm increased markedly and Raman spectroscopy showed a disulfide bridge collapse associated with DNA. The specific activities of CAT and GPX increased in the overweight and obesity groups, but those of SOD did not change. The amounts of proteins in the testes and epididymides decreased. These findings confirm that overweight and obesity decrease concentrations of free testosterone and seem to decrease protein content, causing poor sperm quality. *Implications*. An increase in scrotal fat in these conditions fosters an increase of ROS, but the increase of GPX and CAT activity seems to avoid oxidative stress increase in the sperm without damaging your DNA.

## 1. Introduction

Obesity is a global health problem that has been increasing in recent years. It is associated with hypertension [1], cancer [2], diabetes [3], metabolic disorders [4], and male infertility [5]. Infertility due to a high body mass index (BMI)—above 25–30 kg/m^2^—is associated with decreased sperm concentration and viability, increased morphological abnormalities [6, 7], hormonal alterations [8, 9], modified GnRH pulsatility, altered leptin, insulin, cholesterol, and triglyceride levels, high estradiol levels, and decreased testosterone (T) concentrations [8–10]. These effects alter testicular and epididymal functions and T-dependent organs [11, 12], since one of testosterone's main functions is to induce protein synthesis for the processes of spermatogenesis and sperm maturation and survival [10, 11, 13]. One of the changes in epididymal maturation is the oxidation of SH groups to S-S, generally by ROS [14].

Sperm are at serious risk from the moment they form in the testicles and during transit through the epididymides, since the processes in which they participate are highly sensitive to ROS, especially superoxide and hydrogen peroxide [15], substances that can cause membrane damage and DNA fragmentation in sperm.

Obesity can trigger systemic oxidative stress [16] in the testicles and sperm that can reduce T synthesis, spermatogenesis, and sperm quality [17–21]. In addition, higher amounts of adipose tissue in the scrotal area have been associated with increased oxidative stress [22]. Oxidative stress is caused by an imbalance between ROS production and the activity of three anti-ROS enzymes: SOD, CAT, and GPX. When the amounts of ROS are excessive, cell damage ensues [23]. The presence of the antioxidant enzymes SOD, CAT, and GPX in the reproductive tract of rats has been reported, with high concentrations of GPX in the testes upon reaching sexual maturity but low levels in the epididymides. However, it has been suggested that these enzymes also participate in spermatogenesis and protect the spermatozoa during their transit through the epididymides, from *caput* to *cauda* [12, 24, 25].

SOD is found in extracellular tissues and fluids from the epididymides, prostate gland, and bladder of different animal models (mouse, pig, and boar) [26–28]. In the rat's epididymides, increased SOD and GPX activity has been observed in the *caput* and *cauda* [17, 19].

GPX is one of the most important enzymes in the antioxidant system of the male reproductive tract because its concentration in the testicular epithelium is higher than that of the other antioxidant enzymes [29]. Especially in the epididymis, GPX (snGPX4 and GPX5 in rodents or snGPX4 and PRDX in humans) is very important to achieve optimal sperm maturation [30]. In summary, oxidative processes regulated by GPX are necessary to generate a properly condensed mature sperm nucleus [7]. In addition, as many as 4 isoforms of GPX are associated with the epididymal fluid [31]. GPX-1 and GPX-3 are present in the testes and epididymides but in the latter are expressed mainly in the *cauda* [1, 32]. GPX 1, 3, 4, and 5 are all found in the epithelial cells of the epididymides, luminal fluid, and sperm [31, 33]. GPx-4 is influenced by testosterone since in rat testes it is expressed after puberty and is necessary for germ cell differentiation; hence, it may be involved in spermatogenesis [34].

CAT activity has been found in the *caput* and *cauda* of the epididymis [19]. It is also expressed in Leydig, peritubular myoid, and Sertoli cells and in spermatogonia, though expression is low in spermatocytes and spermatogonia during spermatogenesis [35–37]. It is present, as well, in the epithelium of the epididymis, though at very low levels, and in spermatozoa [19].

Reports in humans indicate the presence of antioxidant enzymes in semen and have found that when the normal concentrations of these enzymes are altered, sperm parameters (motility, viability, and acrosomal reaction capacity) are affected [38–40].

The condition of obesity is defined as a state of acute chronic inflammation that favors an oxidizing environment, so it has been directly associated with infertility in obese individuals [41, 42]. Body weight and length are used to determine obesity in rats by calculating the Lee index, which is analogous to the BMI in humans [8]. This index makes it possible to estimate body composition in rats [43]. It categorizes values *<0.300* as normal weight but values *>0.300* as obesity [6, 43–47].

Given this background, the aim of this work was to determine whether some male fertility problems that occur in overweight and obesity conditions are attributable to an alteration in the activity of the antioxidant enzymes SOD, GPX, and CAT in the testes and epididymides.

## 2. Materials and Methods

All experiments were conducted according to the Mexican Guidelines for Animal Handling and Protection (NOM.062.ZOO 1999). The rats were obtained from the closing breeding colony at the Metropolitan Autonomous University, Iztapalapa Campus. The experimental protocol was approved by the Institutional Animal Care and Use Committee. Proper animal handling was performed in accordance with the Ethical, Science, Communication, and Research specifications established by the Division of Biological and Health Sciences, UAMI (dictum: 1851), and the Organization for Animal Health (OIE) guidelines stipulated in the Terrestrial Animal Health Code, chapter 7.8.

### 2.1. Animals

The study was performed with 3-month-old male Wistar rats which were housed individually in 6 mm crystal acrylic boxes with galvanized and stainless steel lids, a parasite-free pine sawdust bedding and a 250 ml polypropylene drinker were used under controlled light conditions with ad libitum access to regular or high-fat-diet feed and tap water. The animals (*n* = 6 rats per group) were assigned to one of three groups: (1) control (CN: regular chow; 4.5% lard); (2) overweight (OW: high-fat diet; 10% lard); and (3) obese (OB: very high-fat diet; 60% lard) (LabDiet and TestDiet, St. Louis, MO). All animals had free access to food for four weeks. Body weight was measured every day at the same hour. It is important to mention that the initial weight of the animals used, which determine that they have reached adulthood and are in a normal weight condition, was 310 ± 5.066. The measures used to calculate OW or OB were body weight (g), snout-anus length (cm), and the Lee index (cube root of body weight (g) divided by the snout-anus length (SAL) (cm)) [43].

### 2.2. Fertility Analysis

Each male in each group mated with two proven fertile females. The test was performed before and after inducing overweight and obesity, with each male being its own witness. All males were sexually skilled, and all females were in estrus. The intercourse schedule was always between 3 and 4 in the afternoon, and mating was confirmed by the presence or absence of the vaginal plug. After successful mating, males were separated, and that day was considered as day zero of gestation (GD 0) for the females and count 21 for spontaneous delivery. Litter size and sex of the progeny were examined 4 days after birth [48].

At the end of the four-week test period, the rats were euthanized by decapitation and their organs dissected. The fat located around the testis and mesenteric fat was weighed. Blood was collected and centrifuged at 1500 × g for 15 min. Serum was stored at −20°C for later processing. The fat surrounding the epididymides (right and left) was removed and weighed. The epididymides were regionalized into three sections: *caput*, *corpus,* and *cauda*, and each section was divided into two parts, one of which was squeezed to obtain spermatozoa to evaluate viability and morphology and determine DNA damage and reactive oxygen species (ROS). Another section was weighed, frozen in liquid nitrogen (−196°C), and used for the enzymatic determination.

The segments of the epididymides were thawed to 4°C in 10 volumes of cold HEPES buffer (0.1 mol/L, pH 7.4; H0887 Sigma Darmstadt, Germany) and homogenized in a Potter-Elvehjem type, glass-glass homogenizer. The corresponding segments of both epididymides from each rat were always homogenized together. The homogenates obtained were centrifuged for 10 min at 5000 × g. Precipitates were discarded, and all enzymatic activity was measured using the supernatants.

### 2.3. Testosterone Serum Levels

Testosterone levels were measured by radioimmunoassay (RIA) using a commercial kit TKTT-1 (6550-01-288–7029, Siemens Healthcare Diagnostics Inc, Los Angeles, CA). The procedure used antibody-coated tubes in which ^125^I-labeled testosterone competed with free testosterone for antibody sites in the sample. After incubation, separation of bound *T* was achieved by decanting. The tubes were then counted in a gamma counter (Cobra, Packard, USA), and the counts were inversely related to the amount of *T* present in the serum. Total quantities of T in (pg/ml) were determined by comparing counts to a calibration curve. Specific activity was 4 *μ*ci. The sensitivity limit was 0.0045 ng/ml. All measures were made in duplicate.

### 2.4. Sperm Parameters

Each region of the epididymides was rinsed with Ringer physiological solution (95 mM NaCl, 5 mM KCl, 1.7 mM CaCl_2_.2H_2_O, 1.19 mM MgSO_4_.7H_2_O, 1.19 mM KH_2_PO_4_, Sigma Aldrich, México) at 37°C and squeezed with small tweezers to remove the spermatozoa. 10 *μ*l of the solution was placed on a glass slide stained with eosin-nigrosin for 1 minute at 37°C. Approximately 100 cells were counted using a light microscope with a 40 X objective (Opstisum, New York, USA).

### 2.5. Analysis of DNA Integrity

Samples of spermatozoa in a physiological solution were used to evaluate DNA integrity. Sperm smears were spread on precleaned slides and allowed to air dry. The slides were fixed with Carnoy's solution (methanol/glacial acetic acid 3/1; 64-56-1, QUÍMICA UNIVERSAL LTDA, Santiago, Chile/64-19-7, ISQUISA CORPORATIVO, Córdoba Veracruz, México) for 24 hours. Once dry, they were stained with freshly prepared acridine orange stain (65-61-2, Acridine Orange solution, Sigma Aldrich, México) for 5 minutes and then rinsed with distilled water. The samples were read on the same day under epifluorescence microscopy (490 nm) with a 100X objective. One hundred cells were counted, observing the number of green and red fluorescing sperm heads.

### 2.6. Measurement of ROS Production

ROS production was determined by flow cytometry analysis (FACSCalibur, Beckton Dickinson, San Jose, CA) using dichlorofluorescein diacetate (DCF, 4091-99-0, Sigma Aldrich, México), as described previously [24, 49, 50]. In brief, samples with 0.5 × 10^6^ spermatozoa from each group were incubated with 1 mL of Ringer solution and 50 *μ*L of DCF at 32 *μ*M for 15 minutes under dark light at room temperature and then centrifuged for 5 min at 1500 × g. The pellet obtained was dissolved in 1 mL of PBS and analyzed by CELLQUEST software.

### 2.7. Antioxidant Enzyme Activity

Before determining GPX (glutathione peroxidase), SOD (superoxidase dismutase), and CAT (catalase) activity, the organs were thawed to 4°C in 10 volumes of cold HEPES buffer (0.1 M, pH 7.4) and homogenized in a Potter-Elvehjem type, glass-glass homogenizer. The homogenates obtained were centrifuged for 10 min at 5000 × g. The precipitates were discarded, and all enzymatic activity was measured in the supernatants. The assays to quantify enzymatic activity were done as described previously [15].

- GPX (EC.I.II.I.9) activity was determined at 25°C using Ransel kits (RS504, Randox Laboratories Limited, Crumlin, Northern Ireland) to determine NADPH oxidation at 340 nm for 5 min [51].
- SOD (EC.I.15.1.1) activity was measured using RANSOD kits (SD125, Randox Laboratories) at 37°C. Absorbance was monitored at 505 nm for 3 min.
- CAT (EC1.11.1.6) activity was determined at 25°C after pretreatment of samples with ethanol (64-17-5, 0.01 mL EtOH/mL of homogenate) and Triton X-100 (1.0%) (v/v final concentration; 9036-19-5, EMPROVE EXPERT Ph Eur, Sigma Aldrich, México) in an ice bath [52]. After incubation, the amount of H_2_O_2_ (7722-84-1, Millipore Sigma, Darmstadt, Germany) that remained in the mixture was determined by measuring unreacted KMnO^4^ (7722-64-7, Merck, Darmstadt, Germany) at 25°C and 480 nm.

Enzymatic activity was expressed as follows: for superoxide dismutase, in USODs (one unit of activity defined as the amount of enzyme that inhibited the rate of formation of the formazan dye by 50%) [53]; catalase, as the change in k (the constant rate of the first-order reaction) [54]; and glutathione peroxidase, as AGPX (nmol NADPH oxidized per minute).

### 2.8. Quantification of Total Soluble Proteins

Total protein content was measured using a commercially available bi-cinchoninic acid protein assay (23225, Pierce Thermo Scientific, Rockford, IL, USA). All assays were run in duplicate using single and double amounts of homogenate (6 testicles and 6 epididymides were used for the homogenate) [15].

### 2.9. Raman Spectroscopy

Sperm were subjected to a density gradient centrifugation for 5 minutes at 1500 g. After removal of the supernatant, the pellet was resuspended in physiological Ringer solution pH 7.2. The sperm were fixed with paraformaldehyde 2%. The samples were spread on slides and allowed to air dry. RAMAN evaluation were performed on a T64000 Horiba-Jobin-Yvon Lab Ram HM 800 Micro Raman System (HORIBA, Kyoto, Japan) triple spectrometer using a laser line at 532.1 nm produced by a Ventus laser. A confocal microscope Olympus, BX40 (Edison, NJ, USA), with ×100 objective was used at a power closed to 7 mW on the sample, resulting in a typical coverage of about 1 sperm cell. 20 accumulations of 10 s per spectrum were performed in order to improve the signal to noise ratio. The spectra were calibrated using the 521 cm^−1^ line of monocrystalline silicon. Téllez-Plancarte et al., Nazarenko et al.

### 2.10. Statistical Analyses

Data are presented as mean ± standard deviation (mean ± SD), analyzed using omnibus normality and modified Levene equal variance tests and a one-way analysis of variance (ANOVA). Parametric Tukey–Kramer or non-parametric Kruskal–Wallis tests were applied with their respective post hoc tests. A probability value of *P* ≤ 0.05 was considered statistically significant. All statistical analyses were performed with NCSS 2007 DATA Software (Kaysville, UT, USA).

## 3. Results

### 3.1. Fertility Analysis

We also observed that control rats produced 11.8 ± 1.6 pups per litter, while the 10%-lard group produced only 3 ± 3 pups per litter. Surprisingly, in 60% rats, the number of pups per liter was closer to the control (10 ± 0 pups per litter). Gender distribution in the control litter was 3.8 ± 1.3 females and 7.8 ± 2.2 males, while that in the 10% lard group was 2 ± 2 females and 1 ± 1 males. Finally, that in the 60% lard group was 5.5 ± 0.5 females and 4.5 ± 0.5 males (Table 1).

### 3.2. Weight Gain, Scrotal Fat Gain, and Serum Testosterone Levels

**Figure 1:**
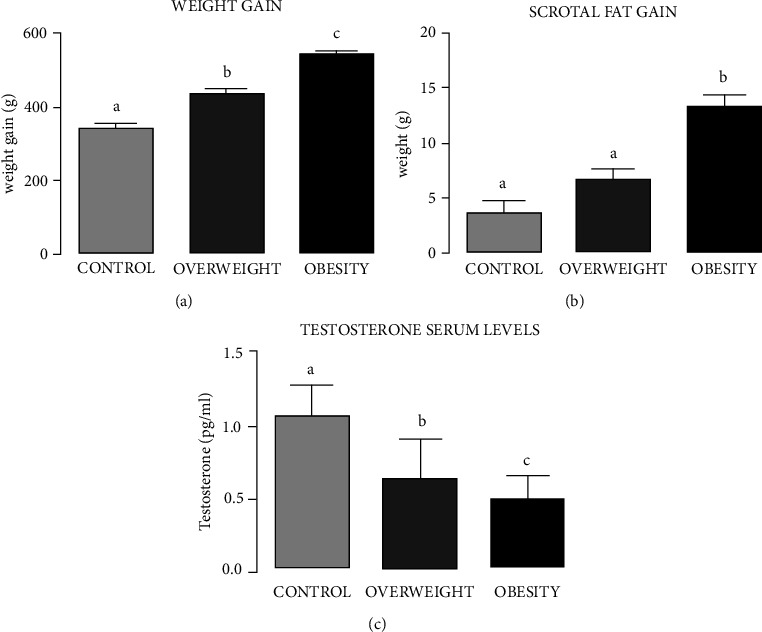
Weight gain, scrotal fat gain, and testosterone serum levels in Wistar rats fed for four weeks with regular chow (control group (CN)) or hyperlipidemic diets with 10% (overweight group (OW)) and 60% fat (obesity group (OB)). Bars represent mean ± SD. Letters indicate significant differences between groups, *P* < 0.05 (CN vs. OW vs. OB).

Body and scrotal fat weight measurements are shown in Figure 1. Testosterone serum levels shown in this figure confirm that gaining weight and fat around the gonads is negatively correlated with testosterone levels. The OW and OB groups had weight increases of 24.17% and 51.12%, respectively, with respect to CN. The weight increase in OB was 29.84% greater than that in OW which also shows that the OW and OB groups had increases in scrotal fat compared to CN (Figure 1). The Lee index for the experimental groups fed regular chow and the two hyperlipidemic diets was 0.329 for OW, 0.339 for OB, and 0.2915 for CN.

Serum testosterone levels (Figure 1) in the OW and OB groups (0.635 pg/ml ± 0.27; 0.502 pg/ml ± 0.15, respectively) decreased significantly compared to CN (1.078 pg/ml ± 0.21).

### 3.3. Quantification of Proteins in the Testicles and Epididymides

**Figure 2:**
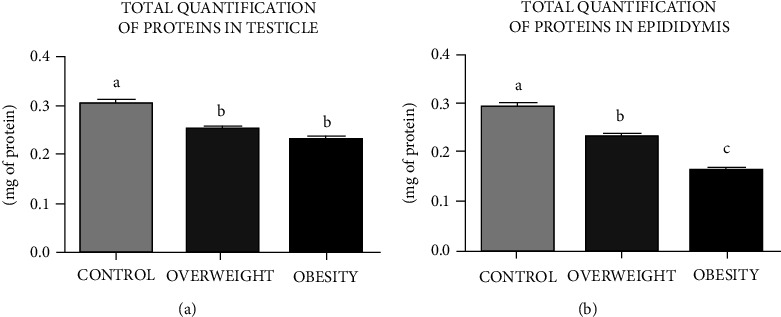
Amount of total soluble protein in testis and epididymis in Wistar rats fed for four weeks with regular chow (control group (CN)) or hyperlipidemic diets with 10% (overweight group (OW)) and 60% fat (obesity group (OB)). Bars represent mean ± SD. Letters indicate significant differences between groups, *P* < 0.05 (CN vs. OW vs. OB).

Protein concentrations in the rats' testicular and epididymal tissue (Figure 2) for groups CN, OW, and OB showed that OW and OB had lower concentrations than CN.

### 3.4. Specific Activity of Proteins in the Testicles and Epididymides

**Figure 3:**
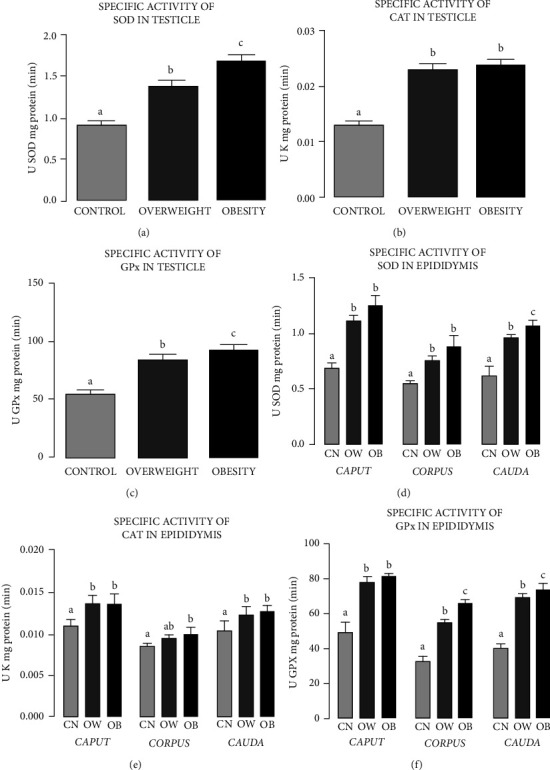
Specific activity (SA) of the antioxidant enzymes superoxide dismutase (SOD), catalase (CAT), and glutathione peroxidase (GPX) in the testes and epididymides of Wistar rats fed with regular chow (control (CN)) or hyperlipidemic diets with 10% or 60% fat (the overweight (OW) and obesity (OB) groups), respectively. Specific activity is expressed in mg of protein/min. Letters indicate significant differences between the study groups (*P* < 0.05) (CN vs. OW vs. OB). Also shown are the SAs of the antioxidant enzymes superoxide dismutase (SOD) (a), catalase (CAT) (b), and glutathione peroxidase (GPX) (c) in the testicles, all presented as mean ± SD.

Results for the specific activity (SA) of the antioxidant enzyme SOD in the testes (Figure 3(a)) of the rats in the CN, OW, and OB groups showed an increase in the testicular tissue for OW and OB compared to CN. SA was greater in the *caput* and *cauda* than the *corpus*, but the difference between *caput* and *corpus* was not significant. In all three regions, the SA of SOD was greater in OW and OB than CN. Regarding the SA of CAT in the testicular tissue (Figure 3(b)) for the three study groups, OW and OB showed increases with respect to CN. Finally, the SA of the GPX enzyme in the testicular tissue of the rats (Figure 3(c)) increased in OW and OB compared to CN.

With respect to the epididymal tissue, there was greater SA in the *caput* and *cauda* than the *corpus* (Figures 3(d)–3(f)), with no significant difference between the *caput* and *corpus*. In the three regions of the epididymides, the SA of SOD was greater in OW and OB than CN. The SA of CAT in the epididymal tissue (Figure 3(e)) also increased in OW and OB compared to CN. In addition, the *caput*, *corpus*, and *cauda* all showed increases in the SA of CAT in OW and OB compared to CN. In this case, the *caput* and *cauda* had higher SA of CAT than the *corpus*. Finally, the epididymal tissue (Figure 3(f)) showed greater SA of GPX in OW and OB than CN, with the *caput* and *cauda* having greater SA of GPX than the *corpus*.

### 3.5. Sperm Parameters

Table 2 represents the sperm parameters evaluated for the three groups of Wistar rats (CN, OW, and OB); viability and morphological abnormalities (amorphous head, angled middle piece, asymmetric middle piece, angled flagellum, rolled flagellum, and coiled). The OW and OB groups had decreased viability and an increased number of abnormalities compared to CN. There was also an increase in morphological abnormalities (angled middle piece, angled flagellum, and coiled) in the sperm in OW and OB compared to CN.

Table 2 shows sperm parameters in Wistar rats fed with regular chow (CN group) or hyperlipidemic diets with 10% or 60% fat (OW and OB groups). Results for the three study groups are shown as mean ± SD. Letters indicate significant differences between the groups, *P* < 0.05 (CN vs. OW vs. OB).

### 3.6. Production of Reactive Oxygen Species

**Figure 4:**
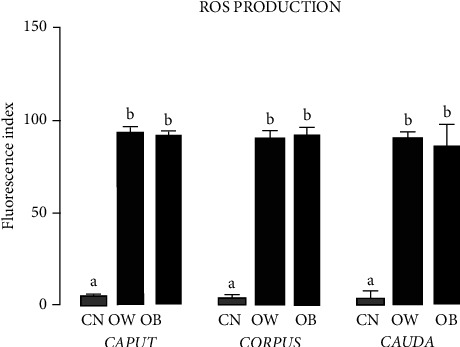
ROS production in the plasmatic membrane of the spermatozoa in the *caput*, *corpus,* and *cauda* regions of the epididymides of the rats fed for four weeks with regular chow (control group (CN)) or hyperlipidemic diets (overweight (OW) and obese (OB) groups). Bars represent mean ± SD. Letters show significant differences between the groups, *P* < 0.05 (CN vs. OW vs. OB). Fluorescence index = (average) (number of events).

Figure 4 shows the results for the peroxide content determined in the epididymal sperm obtained from the *caput*, *corpus*, and *cauda* regions of the rats in the three study groups (CN, OW, and OB). The amounts of peroxides present in OW and OB were larger than those in CN.

### 3.7. Sperm DNA Integrity

**Figure 5:**
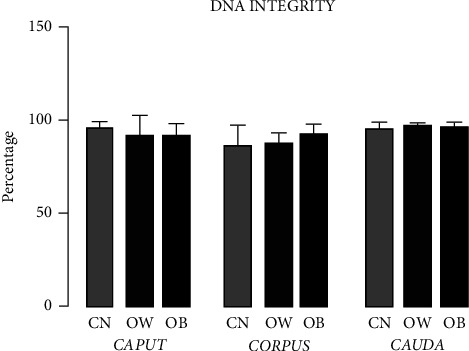
Sperm DNA integrity in the *caput*, *corpus,* and *cauda* regions of the epididymis of Wistar rats fed for four weeks with regular chow (control group (CN)) or hyperlipidemic diets (overweight (OW) and obese (OB) groups). Bars represent mean ± SD.

Figure 5 shows that there is no damage to the integrity of the DNA in the spermatozoa obtained from the three regions of the epididymis observed in the OW and OB groups compared to CN.

### 3.8. Raman Spectroscopy

**Figure 6:**
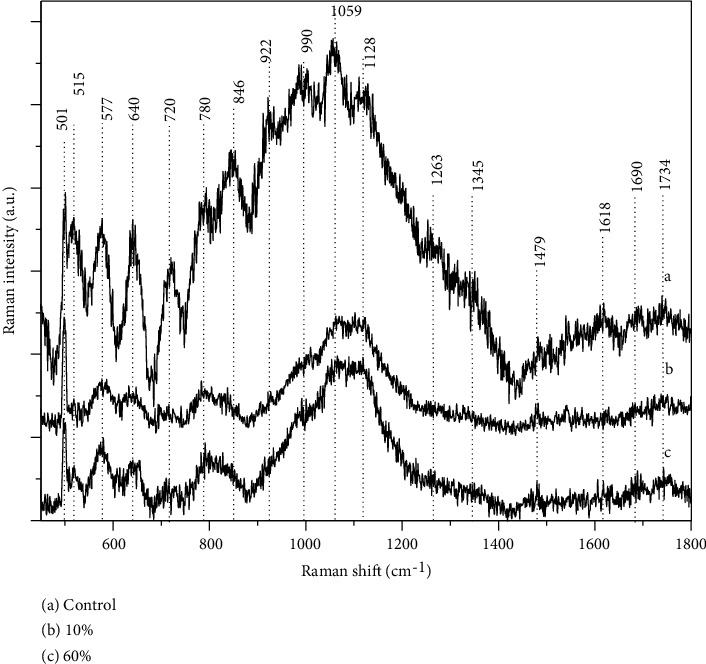
Vibrational information of the chemical bonds of the molecules of the sperm head in Wistar rat in the following groups: (a) control (CN) regular chow, (b) 10% (OW) (c) 60% (OB) lard chow groups.

The Raman spectra results are shown in Figure 6. The spectrum of the control sample displays many bands corresponding to lipids, proteins, and the nucleus of the sperm head (Table 3). Many of these bands have an intensity comparable to the S-S stretching of the disulfide bridge at 501 cm^−1^. The most important change we observed in the sperm spectra of rats fed with 10 and 60% extra fat was the collapse of the bands, mainly those of DNA and proteins, and lipid bands too (1059 and 1128 cm) (Figures 6(b) and 6(c)).

## 4. Discussion

Several studies have reported an association between obesity and reduced fertility including indications of an inverse relationship between the BMI and fertility [55, 56]. Our work found that high-fat diets induced an increase in body weight, as several other researchers have reported [18, 57–59], together with reduced sperm quality that was dependent on the diet administered.

Most of those studies, however, focused only on OB animals. To the best of our knowledge, few have analyzed overweight (OW) animals. Using a rat model with a 10% hyperlipidemic diet, Wood *et al* [20] determined weight increases of 10–25% compared to a group that received a balanced diet. This weight gain indicates an overweight condition or moderate obesity. The rats in their study that received a hyperlipidemic diet with 60–75% fat, in contrast, showed a weight increase greater than 40%, indicative of a state of severe obesity [57, 58].

In our study, the rats with the high-fat diet (10%) showed a body weight increase of 24.17%, which was considered as OW. The rats that received the 60% very high-fat diet for four weeks increased their weight by 51.12% compared to the CN group. This was classified as an OB condition. These data agree with the classification presented in Wood *et al*. [20] However, as in humans, the body mass index is only a reference, their muscular constitution also influences. In the same way, the Lee index is also a reference.

Several authors have reported Lee index values as indicators of obesity that vary with the strain and age of the animal, as well as the time of administration of the diet. Unfortunately, those studies do not provide data regarding OW. Vigueras-Villaseñor *et al.*, for example, administered a hypercaloric diet to 3 month-old Sprague-Dawley rats for 30 days. They reported a Lee index value of 0.3, while the study by Leopoldo *et al.* calculated a Lee index of 0.7 in 30 day-old male Theiller mice fed a hyperlipidemic diet for 15 weeks. In that case, the Lee index was similar in the OW and OB groups (OW = 0.329, OB = 0.339) and insufficient to characterize or differentiate OW and OB [44].

Some studies suggest the importance of considering the correlation between the Lee index and animals' fat mass in order to estimate the body index in rats [44, 46]. Those reports suggest that the Lee index alone should not be used as a reference to determine obesity since in some cases there is no direct correlation between body weight and this index [60]. This occurred in our results, where the OW and OB rats had similar Lee indices, while differential body weight and scrotal fat were observed only in the OB group.

We found, as well, that the high-fat diet (10%) was sufficient to alter spermatogenesis, as has been reported previously by Vigueras et al. and Yang. This process is affected by the degree of obesity [61]. A similar effect was observed in the OB rats as they showed significantly lower sperm quality and an even larger increase in sperm abnormalities than the OW subjects.

The present study found that both OW and OB (fed high-fat diets, respectively) presented decreased testosterone levels, as has been reported for OB animals in various other studies [47, 62–64]. These reduced T levels were related to the degree of obesity and to an increase in estrone and estradiol levels due to peripheral aromatization of androgens that induces endocrine dysregulation, leading to low T levels [65, 66].

Interestingly, the OW rats also had reduced *T* concentrations, as has been reported in humans with OW and OB [67]. This decrease in *T* affects the functionality of the testes and epididymides, both of which are T-dependent organs [14] that function to induce protein synthesis for the various processes involved in spermatogenesis and sperm maturation and survival [14, 68]. In the present case, the OW and OB rats also had a reduction in total protein quantification in both the testes and epididymides that could impact sperm morphology and generate changes in spermatogenesis and epididymal sperm maturation that would result in poor sperm quality [5, 34, 69–71] induced by the reduction in protein synthesis caused by low T concentrations [2, 72].

Several studies have reported that obesity can affect sperm quality. It has previously been reported that at least two generations of males that come from obese parents (the result of consuming a hypercaloric diet) present a large number of chronic diseases such as obesity, type 2 diabetes, and behavioral and reproductive disorders [73, 74]. However, in the present work, an immediate effect is observed, presenting a decrease in the size of the litters, of overweight parents, in addition to a lower proportion of males, which could be explained by a greater vulnerability in male gametes to the effects caused by overweight and obesity. We observed that our OW and OB rats presented reduced sperm quality. Certain mechanisms have been shown to participate in sperm quality due to obesity, including a malfunctioning of the hypothalamic-pituitary-testicular (HPT) axis, low FSH and LH concentrations [75], and alterations in SHBG synthesis [76] that cause an increase of free testosterone, making it available for conversion to E2, a hormone that inhibits GnRH secretion in the hypothalamus [49]. This reduces the concentration of *T* which, in turn, affects the functionality of the Leydig cells [11]. Furthermore, the accumulation of saturated fats in testicular cells like the Sertoli cells alters spermatogenesis due to modifications of the composition of fatty acids in the membrane [11, 77].

It is important to note that these alterations in sperm quality were significantly greater in the OB group than the OW rats, a difference that could be due, as well, to the reduction in T, which alters sperm differentiation and survival [17]. The increase in scrotal fat may also play a role, as it was significantly higher in the OB rats than the OW group. This accumulation of fat increases scrotal temperature in the areas surrounding the testicles and epididymides, conditions that generate scrotal hyperthermia, which is a risk factor for male fertility and has a deleterious effect on spermatogenesis [78, 79].

In this regard, studies have suggested that the excessive presence of fat around the testicles and epididymides in animal and human models of obesity can alter the temperature of these organs, triggering a process likely developed by an increase in the adipose tissue that covers the pampiniform plexus and affects the testicular cooling system [80]. This increase in scrotal temperature induces a series of alterations at the testicular level that are associated with increased apoptosis and a decrease in the number of spermatogonia in the germinal epithelium [47, 81].

Obesity can also cause systemic oxidative stress [13] in the testicles and sperm that reduces testosterone synthesis, spermatogenesis, and sperm quality [4, 82]. Increased adipose tissue in the scrotal area has also been associated with greater oxidative stress that can impact testicular and epididymal functioning [83–86] and sperm quality. In the event of excessive oxidative stress, this nuclear maturation can be impaired, resulting in excessive protamine disulfide bridges, abasic sites (which in themselves do not constitute a DNA break), cross-linking of nuclear proteins, and finally DNA breaks (only when oxidative stress is severe) [87–89]. Sperm are highly sensitive, especially to superoxide and hydrogen peroxide [3, 15, 89], so exposure can generate membrane damage and DNA fragmentation. Potential mechanisms that explain how ROS cause DNA damage are (1) the generation of new molecules from lipids degradation, specifically, malondialdehyde that cause oxidation of the nitrogenous bases of the DNA to produce 8′-hydroxyguanosine (promutagen) or (2) direct interaction of ROS with DNA inducing breaks in one or two chains. However, our results obtained with acridine orange do not expose breaks of DNA chains. Nevertheless, the damage to sperm in overweight and obesity conditions observed through RAMAN spectroscopy, include disulfide bonds collapse of proteins associated with DNA. The use of Raman spectroscopy allowed us to perform an in vivo analysis of the sperm plasma membrane and determine if there is damage to it or if the damage exists at the genome level of sperm from rats exposed to a high-fat diet.

The use of Raman spectroscopy allowed us to perform a live analysis of the plasma membrane and determine whether there is damage to it or to at the genome level of the spermatozoa of rats exposed to a high-fat diet. It is well known that disulfide bridges of DNA associated proteins in rats that were subjected to a high-fat diet (10% and 60%) showed a similar spectrum (501 cm^−1^) relative to animals that were fed a control (low fat) diet. It makes us think that the S-S bonds of the proteins that maintain the adequate packaging of the DNA chain in the sperm are not affected. However, the decrease in the intensity of the DNA spectrum (720–780 cm^−1^), although it may indicate damage, does not seem to be through ROS since it has been reported that ROS oxidize cysteine residues forming disulfide bridges, and according to what was obtained in the present work, there is a collapse in disulfide bridges which may indicate that cysteine residues remain in a reduced state.

The results of this study show that rats with OW and OB present increases in the activity of the enzymes SOD, CAT, and GPX that are similar in the testes and epididymides, together with an increase in ROS in the epididymis, though this did not affect the DNA integrity.

Only the OB rats presented an increase in scrotal fat compared to the CN group, so this increase in the activity of antioxidant enzymes could be due to the decrease in T that was observed in both groups. In this regard, several researchers have reported that testosterone plays a pro-oxidant role and induces oxidative stress in mammalian tissues [90]. However, there are also reports that T has an antioxidant effect on the human prostate gland [91] and the nervous system in rats [16, 92]. Low T levels increase oxidative stress [93] and lead to a redox imbalance [64].

In this regard, Choobineh et al. reported that administering T to mice that presented decreased sperm quality and increased oxidative stress induced by spinal cord injury reduced malondialdehyde levels and SOD and GPX activity in the testicles. In addition, recent data suggest that T exerts antioxidant effects, particularly on the aging process, through an AR-independent mechanism. Though still controversial, the proposed mechanism is based on the conversion of testosterone to estradiol that, in turn, increases the levels of antioxidant enzymes and reduces oxidative stress by genomic and non-genomic mechanisms [28, 94, 95].

Some data show that testosterone produced by aromatization via 17 *β*-estradiol positively regulates the expression and activity of the antioxidant enzymes SOD and GSH-PX while decreasing lipid peroxidation (a marker of oxidative damage) in isolated adult murine cardiomyocytes [21, 96].

## 5. Conclusions

The testes and epididymides are T-dependent organs, so the decrease in testosterone concentrations due to the effect of OW and OB can induce oxidative stress and an increase in the activity of antioxidant enzymes that counteract this process. By being able to contain this process, the DNA is not affected. So, we think that other physiological processes dependent on testosterone may be affected by these conditions of OW and OB, avoiding optimal functionality of the sperm in cases of infertility.

## Figures and Tables

**Table 1:** Effect of overweight and obesity on the fertility of Wistar rats.

	Control	Overweight	Obesity
Total pups	11.600 ± 1.020	7.800 ± 1.470^*∗*^	9.800 ± 0.748
Number of males	7.400 ± 1.356	3.400 ± 1.020^*∗*^	4.400 ± 0.490^*∗∗*^
Number of females	4 ± 1.225	4.400 ± 0.490	5.400 ± 0.490

^*∗*^Indicates significant differences between the control vs. overweight group. ^*∗∗*^Indicates significant differences between the control vs. obesity group, *P* < 0.05.

**Table 2:** Sperm parameters.

Experimental group	Morphological abnormality of the sperm					
	Viability	Amorphous head	Angle middle piece	Asymmetric middle piece	Rolled flagellum	Angled flagellum
Control	96 ± 3^a^	1 ± 1^a^	7 ± 6^a^	0^a^	13 ± 2^a^	2 ± 2^a^
Overweight	70 ± 10^b^	2 ± 1^b^	19 ± 9^b^	1 ± 1^ab^	45 ± 17^b^	14 ± 15^ab^
Obesity	32 ± 20^c^	3 ± 1^c^	34 ± 9^c^	1 ± 1^b^	54 ± 14^b^	22 ± 20^b^

**Table 3:** Frequencies of the main peaks observed from the spermatozoa head.

Raman peaks (cm^−1^)	Assignment
501	S-S disulfide stretching band
515	Phosphatidylinositol
577	Try, C, G
640	C-S stretching
720	DNA
780	DNA
846	Tyr
922	C-C stretching ring
990	C-C stretching
1059	C-N, C-C stretching lipids, PO_2_^−^ stretching
1128	C-C stretching lipids
1263	Aiii, T,A
1345	A, G
1479	CH_2_ deformation
1618	Tyr, try, phe *C*=*C*
1690	AI
1734	*C*=*O*

## Data Availability

The data that support this study will be shared upon reasonable request to the corresponding author.

## Supplementary Materials

Supplementary Materials Proposed mechanism of sperm dysfunction.
