# Supplementary figures and images for: Sperm Dysfunction in the Testes and Epididymides due to Overweight and Obesity Is Not Caused by Oxidative Stress
Source: Int J Endocrinol. 2022 Oct 10;2022:3734572. doi: 10.1155/2022/3734572 (PMC9576436; doi:10.1155/2022/3734572)

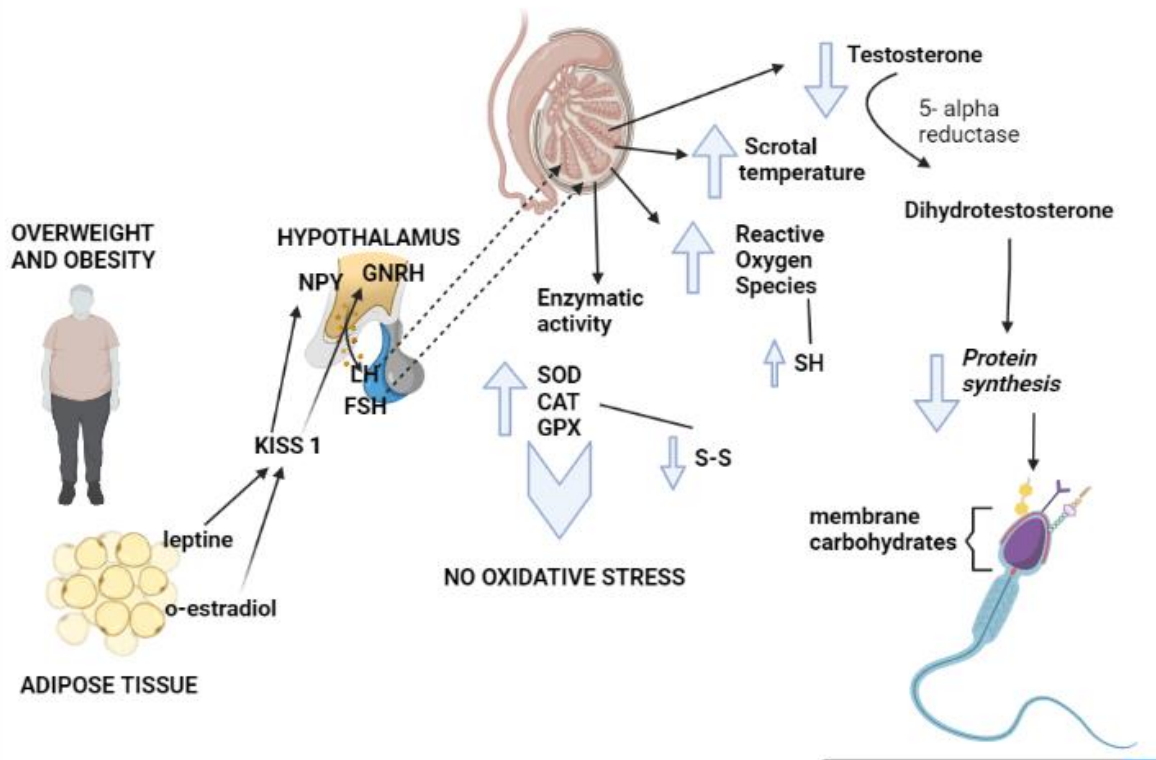

Supplement: Supplementary Materials — Proposed mechanism of sperm dysfunction. [file 3734572.f1.pdf]
